# Correlation between Ethical Intelligence, Quality of Work Life and Caring Behaviour of Paediatric Nurses

**DOI:** 10.1002/nop2.729

**Published:** 2020-12-07

**Authors:** Fateme Shakeri, Foroozan Atashzadeh‐Shoorideh, Maryam Varzeshnejad, Rosana Svetic Cisic, Ber Oomen

**Affiliations:** ^1^ Student Research Committee School of Nursing & Midwifery Shahid Beheshti University of Medical Sciences Tehran Iran; ^2^ Department of Psychiatric Nursing and Management School of Nursing & Midwifery Shahid Beheshti University of Medical Sciences Tehran Iran; ^3^ Department of Pediatric Nursing Nursing & Midwifery School Shahid Beheshti University of Medical Sciences Tehran Iran; ^4^ Post.grad.dipl. King’s college London Zagreb Croatia; ^5^ ESNO, European Specialist Nurses Organization European Public Health Alliance (EPHA) Arnhem the Netherlands

**Keywords:** caring behaviours, ethical intelligence, nurse, paediatric nursing, quality of work life

## Abstract

**Aims:**

The aim of this study is to determine correlation between paediatric nurses' “ethical intelligence” with “quality of work life” and “caring behaviour.”

**Design:**

Descriptive correlational study.

**Methods:**

Data were collected with EIQ, QWL and CBI. Two hundred and one nurses and 201 caregivers of children hospitalized in a paediatric hospital in Tehran were randomly selected as participants. Data were analysed by SPSS. The data were collected in 2019.

**Results:**

Comparison of the subscale “ethical intelligence” with the scale “quality of work life” indicated a significant positive correlation between “honesty” with “job and carrier satisfaction” and “forgiveness” with “job and carrier satisfaction”. In addition, findings showed a significant positive correlation between “honesty” and “control at work” and between “accountability” with “home–work interface.” There was no significant correlation between “ethical intelligence” and “caring behaviours” and between nurses' “quality of work life” and “caring behaviours.” Structural equation modelling showed a correlation between nurses' “ethical intelligence” and “quality of work life.”

## 1 INTRODUCTION

Developing ethical intelligence makes individuals, organizations and social systems more positive and healthy (Ghayumi & Imani, 2015). Studies show that there is a relationship between awareness of professional ethics and compliance with nursing standards in hospitals (Abedi et al., 2015). The quality of care depends largely on the performance of the nurses. Also, Ethics and clinical practice cannot be separated. As a result, nurses' ethical behaviour is considered an effective factor in improving and restoring clients' health (Tanner & Christen, 2014). Assessment of nurses' caring behaviour is different when children are the recipients of care (Salmani et al., 2017). The basic disparities between children and adults, paediatric nursing has its own features that are different from those of adults (Hesanpour et al., 2018). Moreover, nurses' quality of work life influences patient safety and health outcomes both directly and indirectly (Wu et al., 2011). Therefore, emphasizing the promotion of ethical environment through the cultivation of ethical intelligence can eliminate the challenges related to the quality of working life of nurses that affect their caring behaviour.

### 1.1 Background

Nursing care is an intrinsic issue, and it is expected that nurses behave ethically all the time as part of their professional character. It is highly important for nurses to remain sensitive to the consequences of their interventions for patients despite various ethical problems they encounter (Escolar‐Chua, 2018). Nurses' performance is based on humanistic values and the quality of the care provided depends greatly on nurses' performance, ethics cannot be practically separated from clinical practice (Beykmirza et al., 2019). Since nurses form the greatest community in the healthcare system (Pishgooie et al., 2019), their reduced quality of work life may be associated with decreased revenue and organization leave, affecting their occupational performance negatively (Lin et al., 2013). Quality of work life in healthcare organizations has been recognized as a combination of advantages and disadvantages in the work environment (Phan & Vo, 2016). It exerts a significant effect on personnel's behavioural feedbacks such as job and career satisfaction, occupational contribution, professional efforts, occupational fatigue/burn out and organizational change and transfer (Najibi et al., 2017). Factors such as age, gender, work shift, work milieu, occupational satisfaction and work–home equilibrium affect nurses' quality of work life (Lee et al., 2013). According to Said et al, there are six factors related to quality of work life in nursing: Job and career satisfaction, general well‐being, stress at work, control at work, home–work interface and working conditions (Said et al., 2015). Additionally, differences in patient satisfaction with nursing care quality may be attributed to various factors including culture, expectations, previous experiences, personal and social values, life style and patients' awareness of their rights (Shahvali et al., 2018). The basic disparities between children and adults, paediatric nursing has its own features that are different from those of adults. Paediatric nursing is exposed to greater risk of occupational dissatisfaction, emotional stress, diminished mental health and occupational burn out due to exposure to emotional tension of child care whereby affecting nurses' quality of work life (hesanpour et al., 2018).

The results of Braithwaite study also showed that the workload in the neonatal department is effective on job satisfaction, quality of care provided and quality of work life of nurses (Braithwaite, 2008). Also, the result of research in paediatric department showed that the significant positive relationship between quality of work life and quality of nursing care suggests that employees with high quality of work life are able to deliver high quality of care (Mohamed et al., 2016).

Supervision of nurses' work conditions and improvement of organizational conditions of hospitals would predispose to increased patient safety. Managers can influence job satisfaction through affecting the ethical milieu of the organization (Abou Hashish, 2017). Nurses' ethical behaviour is considered as an effective factor in improving and restoring clients' health (Tanner & Christen, 2014). Besides, nurses spend more time with patients compared with other healthcare staff. Thus, they enjoy a unique position in the efficacy and promotion of healthcare services (Shahvali et al., 2018). Given that children are not sufficiently grown up to express their own opinions, parents are their legal decision‐makers bearing the responsibility of qualitative evaluation of the care given to their children. In the other words, parents are rendered as supporters and decision‐makers of hospitalized children and their opinions represent the attitudes of the children. Investigation of opinions of parents of hospitalized children may be considered as a basis for assessing quality care and developing family‐centred care and an effective factor influencing the quality of care (Salmani et al., 2017). Therefore according to the present challenges of nurses' ethical intelligence and its association with quality of work life and quality care and specific sensitivity of the issue in paediatric wards the aim of this study was determine the correlation between nurses' ethical intelligence and quality of work life and between nurses' ethical intelligence and caring behaviours in a paediatric hospital.

## 2 METHODS

### 2.1 Design

The study units in this descriptive correlational study consisted of 201 nurses and 201 caregivers of children hospitalized in a major paediatric hospital in Tehran, capital of Iran. Sample size was calculated in G‐Power software. The samples were selected with random simple sampling method. The attendants of hospitalized children were also selected with random simple sampling based on inclusion criteria and hospitalization ward, provided that parents take care of the child under care of nurses participating in the study.

### 2.2 Measures

Data were collected with the researcher‐made “Demographic Questionnaire” and Lennick and Kiels' Ethical Intelligence Questionnaire that is a 40‐item instrument and uses a 5‐point Likert scale ranging from “never”–“always.” Any of 10 aspects of ethical intelligence are assessed with four items. A score of 90–100 is rendered as excellent, 80–89 as very good, 70–79 as good and 69 and less as weak. The face and content validity and reliability of the instrument have been approved by previous studies (Ghayumi & Imani, 2015; Jirdehi et al., 2018). In the present study, Cronbach's *α* reliability coefficient of the instrument was .92 using internal consistency coefficients. The Nurses' Quality of Work Life Scale is a 24‐item inventory developed by Easton & Van Laar. It includes six subscales and uses a 5‐point Likert scale ranging from “strongly disagree = 1”–“strongly agree = 5.” In this instrument, a score of 23–73 indicates low quality of work life, 74–84 moderate quality of work life and 85–115 high quality of work life. The face and content validity and also reliability of the instrument have been approved in some studies (Duyan et al., 2013; Lin et al., 2013) In this study, Cronbach's *α* coefficient of the tool was .86. Moreover, the 16‐item Wolf et al.'s “Caring Behaviors Checklist” was used with a 5‐point Likert scale. This checklist consists of four subscales of “respect for others,” “ensuring of human presence,” “communication and positive inclination” and “professional knowledge and skills.” The face, content and construct validity and reliability of the instrument were confirmed by previous studies (Asadi & Shams Najafabadi, 2014; Atashzadeh‐Shoorideh et al., 2017; Rafii et al., 2008; Wolf et al., 2017).

### 2.3 Analysis

In the present study, Cronbach's *α* internal consistency coefficient was estimated at .97.

Sampling was done during the morning, afternoon and evening shifts in eight episodes. First, the Ethical Intelligence Questionnaire and Quality of Work Life Questionnaire were distributed among the nurses and the completed questionnaires were collected. In addition, child attendants simultaneously with distribution of nurses' questionnaires completed the Caring Behaviors Checklist. Confounding data were excluded. The sample loss was not significant. In the course of the research, any bias was attempted to avoid. Data were analysed with SPSS21 using descriptive statistics of frequency, mean, *SD*, Spearman correlation coefficient and regression analysis using the step by step method (CI = 95%).

## 3 RESULTS

Demographic variables suggested that 96% of the nurses were female and 4% male. Most nurses (49.3%) were aged between 25–35 years. In addition, 86.6% of them held a BS degree, 58.7% were married and 80.6% had a work experience of 15 years or less. Demographics of child attendants demonstrated that 96% of them were female, 4% were male, 49% belonged to the age group of 31–40 years, 46.5% held a high school diploma and 52.8% reported a moderate economic status. In addition, 57.5% of children had a history of previous hospitalization.

Other results revealed that the overall mean of nurses' ethical intelligence was at the very good level. The mean total score of nurses' quality of work life suggested that it was at the low level. Besides, the mean score of nurses' caring behaviour was at the moderate level (Table 1).

**Table 1:** Mean and standard deviation of variables and subscales

Variable	*X* ± *SD*
Total nurses' ethical intelligence	81.27 ± 7.95
Honesty	62.78 ± 6.32
Accountability	48.70 ± 5.41
Forgiveness	33.23 ± 3.94
Compassion	6.94 ± 2.34
Total nurses' quality of work life	69.62 ± 14.27
General well‐being	19.34 ± 4.41
Home–work interface	8.37 ± 2.85
Job and career satisfaction	18.70 ± 4.12
Control at work	9.45 ± 2.45
Work conditions	8.09 ± 2.75
Stress at work	5.40 ± 1.70
Nurses' caring behaviour	48.34 ± 15.13

A comparison of the subscale's “honesty” and “forgiveness” with the subscale “control at work” (*p* < .05, *r* = .21) and the component “accountability” with “home–work interface” (*p* < .05, *r* = .25) suggested a significant positive correlation. Nevertheless, there was no significant correlation between nurses' ethical intelligence and caring behaviours and between nurses' quality of work life and caring behaviours. Linear regression analysis indicated that ethical intelligence significantly predicted 2.7% of quality of work life; however, it does not predict nurses' caring behaviour (Table 2).

**Table 2:** Linear regression of variables

Predictors	*B*	*SE*	Beta	*T*	*p*
Nurses' Quality of work life (Constant)	50.186	7.365		6.814	.000
Ethical intelligence	0.290	0.090	0.173	3.235	.001*
Notice	*R* ^2^ = .173 *R* = .030 Adj.*R* ^2^ = .027				
Nurses' Caring behaviour (Constant)	48.856	9.977		4.897	.000
Ethical intelligence	−0.009	0.121	−0.004	−0.073	.942
Notice	*R* ^2^ = .004 *R* = .000 Adj.*R* ^2^ = −.004				

* *p*‐value < .05.

## 4 DISCUSSION

In this study, the total mean score of nurses' ethical intelligence was at a very high level. The greatest mean score belonged to “honesty” and the smallest mean pertained to “compassion.” A similar study conducted in 2018 used Ethical Intelligence Questionnaire and reported the nurses' ethical intelligence at the higher than moderate level (Jirdehi et al., 2018). Another similar study (2015) reported nurses' ethical intelligence at a good level (4). Akbarilakeh et al. (2019) also reported nurses' ethical intelligence as appropriate (Akbarilakeh et al., 2019). Besides, Langlois and Lapointe (2007) assessed this variable at an appropriate level (Langlois & Lapointe, 2007), a finding which is consistent with our results. In a review study in New Zealand, Wood (2005) demonstrated that most nurses consider the ethical codes, but they do not possess sufficient power and support to perform suitably; this is not consistent with our findings (Woods, 2005).

It appears that nurses' ethical intelligence is higher than moderate in most studies confirming the high rate of ethical intelligence. This may also be attributed to limitations of the questionnaires as they are based on self‐report because the participants may bias subjectively what is in their minds not what is there and thus confounding the data. Therefore, it appears that teaching ethical intelligence to nurses enhances their cognitive ability to cope with personal and interpersonal problems enabling them to exercise an accurate judgment towards the challenging situation they face before showing any behavioural reaction. Therefore Recommended that ethical intelligence training added to in‐service training courses for nurses because learning happens in different contexts, over the course of a lifetime (http://www.europarl.europa.eu/thinktank/infographics/lifelonglearning/index.html). Another approach recommended is the reinforcement of ethical intelligence through organizational identification, introduction and encouragement of the staff with higher ethical intelligence, which is of utmost significance, specifically in paediatric wards.

Another finding of the present study indicated that the total mean score of paediatric nurses' quality of work life was low. In this regard, the maximal mean pertained to the subscale “general welfare” and the minimal mean belonged to the subscale “stress at work.” In the study by Almalki et al. (2012), performed in Saudi Arabia, the participating nurses generally evaluated their quality of work life at low levels. Furthermore, in the present study, the satisfaction level with the subscale “job and career satisfaction” was low whereas in Almalki et al.'s study, the nurses assessed their satisfaction with nursing as appropriate. In addition, Almalki's study was not consistent with the present study in the subscale “general well‐being” as this subscale obtained the highest score of satisfaction in the present study. Besides, in a consistent manner, the participants in this study and in Almalki's study were not sufficiently satisfied with the subscale “home–work interface” (Almalki et al., 2012). Moreover, the study by Ramesh et al. (2013) reported nurses' dissatisfaction with quality of work life (Ramesh et al., 2013). On the other hand, the study by Karaaslan and Aslan (2019), carried out in Turkey on 224 nurses, reported nurses' quality of work life at the moderate level (Karaaslan & Aslan, 2019). Also, Akter et al. (2018) showed in their study on 288 nurses in Bangladesh that nurses' quality of work life was moderate in all aspects (Akter et al., 2018). Additionally, various studies have expounded on the factors determining quality of work life. Among them, Kelbiso et al. (2017) referred to education level/literacy, monthly income, working ward and work environment as factors that determine nurses' quality of work life (Kelbiso et al., 2017). The study by Devi and Hajamohideen (2018) demonstrated that literacy level, monthly income, working ward and work environment are strong predictors of nurses' quality of work life. The researchers of this study predict that provided healthcare managers consider the key issues on quality of work life, nurses' views of quality of work life would be adjustable (Devi & Hajamohideen, 2018). In the study by Hemanathan et al. (2017), “work conditions” received the lowest score in nurses' perspective as one aspect of nurses' quality of work life resulting from community's weak mentality towards nurses and lack of their occupational security (Hemanathan et al., 2017). Furthermore, the study by Moradi et al. (2014) found a significant positive correlation between “literacy level, work experience and type of hospital as workplace” and “quality of work life” (Moradi et al., 2014). Low quality of work life may be followed by negative consequences as the study by Kaddourah et al. (2018) showed that 94% of nurses, dissatisfied with their quality of work life and decided to change their present workplace (Kaddourah et al., 2018). Hence, it appears generally that nurses' quality of work life has been inappropriate in most studies reviewed. This should be accounted for by managers. Of course, there are disparities with respect to the rate of satisfaction with the subscale “nurses' quality of work life.” This ought to be considered meticulously in policymaking to foster nurses' quality of work life. Additionally, some useful measures ought to be taken to promote nurses' quality of work life including implementing organizational reforms, improving nurses' working conditions before employing sufficient manpower, observing standards of staff's work programme, allocation of suitable financial and welfare facilities. Improving work conditions, facilities and ergonomics, investigating and applying personnel's opinions on quality of work life, creating positive spiritual atmosphere in the work environment regarding justice and equity, reciprocal respect, clear sensible expectations, justified award and punishment system and support for human rights are necessary for improving quality of work life.

Another finding of the present study showed that the nurses' mean score of satisfaction with caring behaviour was moderate. This is consistent with the results of the study by Jirdehi et al. (2018) where 73.8% of the patients reported their satisfaction with caring behaviour at the moderate level (Jirdehi et al., 2018). Nonetheless, Dzomeku et al. (2013), inconsistent with our findings, assessed patients' satisfaction with caring behaviour as weak (Dzomeku et al., 2013). The study by Papastavrou et al. (2012) carried out in Cyprus on nurses' caring behaviour suggested a significant difference between nurses' and patients' perceptions of human presence and respect so that the percentage of negative responses to all items under study was greater for patients compared with nurses (Papastavrou et al., 2012).

Caring behaviours are a set of performances and purposive nursing attitudes aimed at meeting patient needs and alleviating their sufferings. Thus, the better the caring behaviour, the easier it will be to transfer professional competency to clients and hence highlighting nurses' concern for patient comfort. As shown in the study by Chen et al. (2018), caring behaviour directly affects self‐awareness of critical insight and thought. Also, caring behaviour indirectly influences critical thinking through self‐awareness and insight (Chen et al., 2018). On the other hand, many factors may affect nurses' caring behaviour so that the study by Sarafis et al. (2016) suggested that nurses' exposure to stressors is considered as a predictor of nurses' caring behaviour, negatively affecting their quality of work life (Sarafis et al., 2016).

This result indicates that patients' expectations of nurses' caring behaviour in both this study and the reviewed study are much greater than what is already declared. Thus, the care ought to be patient‐centred and based on demographics, interests and cultural, social and physiological features that are unique to each client. Other findings of the study indicated a correlation between nurses' ethical intelligence and quality of work life. Besides, the results of linear regression analysis suggested that ethical intelligence predicted significantly 2.7% of quality of work life. In this line, the study by Panigrahi and Al‐Nashash (2019) revealed that quality of work life is significantly and positively correlated with job satisfaction (Panigrahi & Al‐Nashash, 2019). Another study (2019) on assessing the correlation between ethical intelligence and quality of work life indicated a significant correlation between components of ethical intelligence and components of quality of work life except for “work programme” (Mohamadi et al., 2014). Najibi et al.,(2017) found a significant correlation between all aspects of “quality of work life,” except for safe and healthy work environment, general atmosphere of life and development of human abilities and “ethical intelligence” (Najibi et al., 2017).

The present study showed that ethical intelligence predicts 2.7% of quality of work life. This is not consistent with the study by Mohamadi et al. (2014) who showed that ethical intelligence can predict home–work interface, quality of work life and quality of work background, yet it does not play a significant role in predicting quality of work programme (Mohamadi et al., 2014).

It seems that any investment in promoting ethical intelligence improves the aspects of nurses' quality of work life. It is necessary to pay due attention to nurses' quality of work life as a job with high distress and burnout to support work force, promote care and improve the organization. In addition, findings showed positive significant correlations between components of honesty and forgiveness with the subscale “job and career satisfaction,” between the component “honesty” with the subscale “control at work” and between the component “accountability” and the subscale “home–work interface.” In this regard, Lawler et al. (2005) concluded in their study that forgiveness, as one component of ethical intelligence, affects compatibility with stressful events; thus, the stressful environment of the hospital can negatively affect nurses' quality of work life (Lawler et al., 2005).

Furthermore, MacBeth and Gumley (2012) concluded in their meta‐analysis on components of ethical intelligence that the increased ability for compassion as one component of ethical intelligence leads to individual's improved psychological well‐being that is itself correlated with quality of life and thus influencing it (MacBeth & Gumley, 2012). Cox et al. (2012) also concluded that strong ethical beliefs are associated with greater mental health (Cox et al., 2012).

In this study, there was no significant correlation between nurses' ethical intelligence and its components with nurses' caring behaviour. Indeed, linear regression analysis suggested that ethical intelligence does not significantly predict nurses' caring behaviour. In this line, the study by Jirdehi et al. (2018), aimed at determining the correlation between nurses' ethical intelligence, nurses' performance and patient satisfaction, indicated no statistically significant correlation among these variables. Although ethical intelligence was estimated in the present study to be similar to the study above, the rate of patient satisfaction with nursing care was moderate (Jirdehi et al., 2018). This is consistent with our results. On the other hand, other studies were not consistent with our research, such as the study by Updegraff (2013) that emphasized the positive correlation between ethical intelligence and promotion of nursing care (Updegraff, 2013). Donkor and Andrews (2011) showed that teaching of ethical intelligence to nurses increases quality of care). Rani et al. in Malaysia showed that promotion of ethical intelligence in nursing students would lead to their fostered care‐giving performance (Rani et al., 2013), and Sadeghi et al. showed a significant positive correlation between nurses' ethical intelligence and patient satisfaction with nursing care (Sadeghi et al., 2015). Indeed, in the studies reviewed above, teaching exerted a positive effect on caring behaviour as a subscale of ethical sensitivity whereas in the present study, professional knowledge and skills, as a subscale of caring behaviour, had no significant correlation with nurses' ethical intelligence.

Another finding of the present study showed no significant correlation between nurses' quality of work life and components of nurses' caring behaviour. The study by Hamim et al. (2017) indicated that quality of work life influences nurses' caring behaviour. Strategies for promoting nurses' quality of life predisposes to enhancement of their caring behaviour. Feeling and manifestation of quality of work life by the nurse affect both the nurse and the surrounding milieu (Hamim et al., 2017).

Given that work condition and work environment affect nurses' quality of work life and considering that cultural and organizational factors play a determining role in assessing nurses' quality of work life and caring behaviour, lack of similarity or match in study populations may justify the disparities in results. Path analysis of the three variables under study here was done for the first time, being one strong point of the study. Since path analysis in this study showed that ethical intelligence can predict nurses' quality of work life while it does not affect caring behaviour, other future studies should identify the factors affecting caring behaviour using path analysis. Finally, this study was performed in only one paediatric hospital. This limitation attenuates the generalizability of the findings.

## 5 CONCLUSION

Given the significant correlation between nurses' ethical intelligence and quality of work life and considering low level of nurses' quality of work life in this and similar studies, it may be concluded that any promotion of nurses' ethical intelligence would predispose to an enhancement of their quality of work life. Given that all the nurses in the present study were selected from one hospital, the generalizability of the findings should be with caution. Consequently, it seems mandatory to create the required prerequisites for promoting and substantiating ethical intelligence in nurses to increase patient satisfaction with nursing care leading to nurses' fostered quality of work life.

### 5.1 Limitation

The limitation of the present study was the use of self‐reported questionnaires/tools that may limit the generalization of the results.

## CONFLICT OF INTEREST

The authors declare that they have no conflict of interests.

## AUTHOR CONTRIBUTIONS

All authors (FS, FA, MV, MZ, RSC and BO) have participated in the conception and design, analysis and interpretation of data. FS contributed the data collection. FS, FA, MV, MZ, RSC, BO involved in drafting the manuscript or revising it critically for important intellectual content. All authors read and approved the final manuscript.

## ETHICAL APPROVAL

The research proposal was approved with code of ethics no.: IR.Sbmu.REC.1397.056.

## Data Availability

The corresponding author will provide the data of the article upon request.
